# The efficacy of artificial intelligence in diabetic retinopathy screening: a systematic review and meta-analysis

**DOI:** 10.1186/s40942-025-00670-9

**Published:** 2025-04-22

**Authors:** Abdullah S. Alqahtani, Wasan M. Alshareef, Hanan T. Aljadani, Wesal O. Hawsawi, Marya H. Shaheen

**Affiliations:** 1 Department of Surgery, Division of Ophthalmology, King Abdulaziz Medical City, Ministry of National Guard Health Affairs, Jeddah, Saudi Arabia; 2 King Saud Bin Abdulaziz University for Health Sciences, Jeddah, Saudi Arabia; 3 King Abdullah International Medical Research Center, Jeddah, Saudi Arabia

**Keywords:** Diabetic retinopathy, Artificial intelligence, Screening

## Abstract

**Background:**

To evaluate the efficacy of artificial intelligence (AI) in screening for diabetic retinopathy (DR) using fundus images and optical coherence tomography (OCT) in comparison to traditional screening methods.

**Methods:**

This systematic review was registered with PROSPERO (ID: CRD42024560750). Systematic searches were conducted in PubMed Medline, Cochrane Central, ScienceDirect, and Web of Science using keywords such as “diabetic retinopathy,” “screening,” and “artificial intelligence.” Only studies published in English from 2019 to July 22, 2024, were considered. We also manually reviewed the reference lists of relevant reviews. Two independent reviewers assessed the risk of bias using the QUADAS-2 tool, resolving disagreements through discussion with the principal investigator. Meta-analysis was performed using MetaDiSc software (version 1.4). To calculate combined sensitivity, specificity, summary receiver operating characteristic (SROC) plots, forest plots, and subgroup analyses were performed according to clinician type (ophthalmologists vs. retina specialists) and imaging modality (fundus images vs. fundus images + OCT).

**Results:**

18 studies were included. Meta-analysis showed that AI systems demonstrated superior diagnostic performance compared to doctors, with the pooled sensitivity, specificity, diagnostic odds ratio, and Cochrane Q index of the AI being 0.877, 0.906, 0.94, and 153.79 accordingly. The Fagan nomogram analysis further confirmed the strong diagnostic value of AI. Subgroup analyses revealed that factors like imaging modality, and doctor expertise can influence diagnostic performance.

**Conclusion:**

AI systems have demonstrated strong diagnostic performance in detecting diabetic retinopathy, with sensitivity and specificity comparable to or exceeding traditional clinicians.

**Supplementary Information:**

The online version contains supplementary material available at 10.1186/s40942-025-00670-9.

## Introduction

Diabetes is a major global health issue, affecting an estimated 463 million people worldwide. This number is projected to increase to 700 million by 2045 [1]. Diabetic retinopathy (DR) is a leading cause of vision loss globally, affecting millions of people with diabetes. Early detection and timely intervention are crucial to prevent vision loss [2]. Clinically, DR is defined as a microvascular condition that affects the capillaries of the retina causing damage and secondary visual impairment. The underlying mechanisms involve the long-standing hyperglycemia and its sequels [2, 3]. Among diabetic patients, the global prevalence of DR was 22.27% in 2020 and the number of people with DR was estimated to be 103.12 million worldwide [1]. Traditional screening methods often rely on manual examination by ophthalmologists, which can be time-consuming, resource-intensive, and subject to human error.

Artificial intelligence (AI) has emerged as a promising tool for automating DR screening, offering potential improvements in efficiency, accuracy, and accessibility [4]. Recent advancements in computing power have made deep learning the leading AI technique for DR screening. Many deep learning models have outperformed traditional feature-based machine learning methods [5]. This systematic review and meta-analysis aimed to evaluate the efficacy of AI-based screening for DR using fundus images and optical coherence tomography (OCT) in comparison to traditional methods. By synthesizing the existing evidence, this study seeks to inform healthcare decision-makers about the potential benefits and drawbacks of AI-assisted DR screening and guide future research efforts.

## Materials and methods

### Search strategy

This systematic review was registered with PROSPERO (ID: CRD42024560750). We conducted a systematic review and meta-analysis to determine the efficacy of artificial intelligence in the screening of diabetic retinopathy. A systematic search using PubMed Medline, Central, ScienceDirect, and Web of Science was conducted to identify studies on DR and AI. We used a combination of keywords and Medical Subject Headings (MeSH) terms, including “diabetic retinopathy,” “screening,” “artificial intelligence,” “deep learning,” “machine learning,” and “computer-aided diagnosis.” The search was conducted across all fields, including the title, abstract, and MeSH terms, as outlined in Supplementary Table 1. We included publications in English published up to July 22, 2024. Further literature search consisted of reviewing the reference lists of relevant articles such as previous country or region-based systematic reviews and meta-analyses about DR screening using AI. This adopted strategy identified all articles used in previous reviews. No informed consent was required because of the retrospective nature of the study.

### Study selection and eligibility criteria

Studies that met the following criteria were included: (1) diagnostic accuracy studies; (2) clear definition of random sampling procedure; (3) had a response rate above 60%, to ensure sufficient representation and minimize selection bias; (4) participants ≥ 18 years old; (5) known diagnosis of type 1 or type 2 diabetes mellitus; (6) and Diabetic retinopathy with all stages (mild, moderate, severe). However, we excluded all studies that (1) were duplicates, (2) did not have full-text articles, (3) Patients with other retinal diseases, (4) Persistent retinal impairments other than diabetic retinopathy in one or two eyes, (5) Previous retinal surgeries and interventions, and (6) Patient with contraindication to fundus photography. Four reviewers independently screened titles, abstracts, and full texts for relevance and performed data extraction, using a predefined data collection sheet. Any disagreements were addressed through discussion with the principal investigator.

### Data extraction

After obtaining the complete articles, four reviewers—MS, WA, HA, and WH—independently analyzed the features of the included studies and extracted findings related to the diagnostic effectiveness of AI from each study. Any discrepancies between the reviewers were first discussed among the four to reach a consensus. If disagreements persisted, they were resolved through discussion with a fifth investigator, AA, who acted as an arbitrator to ensure consistency and accuracy in data extraction. The reviewers gathered key indicators, including sensitivity (SE), specificity (SP), the number of patients with diabetic retinopathy (DR), and the overall number of participants from the studies. These indicators were then used to calculate the outcome variables for the diagnostic meta-analysis, which included true positives (TP), false positives (FP), false negatives (FN), and true negatives (TN). The results were compiled into contingency tables for use in the meta-analysis. If a study presented different types of DR or employed various algorithms, leading to multiple contingency tables, we considered these as independent entities.

### Quality assessment

To evaluate the quality of the included studies, two reviewers (WH and WA) utilized the Quality Assessment of Diagnostic Accuracy Studies 2 (QUADAS-2) and RevMan 5.4.1. The QUADAS-2 framework consists of four components for assessing the risk of bias: patient selection, index test, reference standard, and flow and timing. Each component contains two or three pertinent questions. The response to each question was Yes, No, or Unclear; The latter is only used when there is insufficient information to judge. A component was deemed to be at low risk if all responses were “Yes” or at high risk if a response was “ No” to any of the pertinent questions. Furthermore, the elements of patient selection, index test, and reference standard were also assessed regarding their clinical applicability. If these components were found to be of “low risk,” it indicates that the studies included in the review are less likely to be biased.

### Data synthesis and analysis

We utilized MetaDiSc software (version 1.4) and employed a bivariate random-effects model to analyze the outcome variables (True Positives, False Positives, False Negatives, True Negatives). This model was chosen because it accounts for both within- and between-study variability, making it suitable for diagnostic accuracy studies that involve heterogeneous data sources. Unlike univariate models, the bivariate random-effects approach considers the correlation between sensitivity and specificity, allowing for more precise pooled estimates despite variations in study design, AI algorithms, and patient populations.The results were presented using Summary Receiver Operating Characteristic (SROC) plots, along with forest plots and a Fagan nomogram. Furthermore, the bivariate random effects model was used to determine the pooled sensitivity, specificity, area under the curve (AUC), diagnostic odds ratio (DOR), adjusted diagnostic odds ratio (ADOR), and positive and negative likelihood ratios (LR + and LR-, respectively). In addition, we calculate the Q index for both the AI screening method and doctor practices to determine whether AI enhances diagnostic accuracy.

The Fagan nomogram was analyzed along with a positive post-test probability and a negative post-test probability to show how the AI test results modify the probability of the presence of DR. This visualization will help convey the effectiveness of AI in altering diagnostic probabilities compared to traditional methods. Additionally, Subgroup analyses were performed according to clinician type (ophthalmologists vs. retina specialists) and imaging modality (fundus images vs. fundus images + OCT). These specific subgroups were chosen because imaging modality can influence AI diagnostic performance due to variations in image resolution and the depth of structural details captured. Moreover, AI performance may vary depending on whether it is compared to general ophthalmologists or retina specialists, as the latter have more specialized expertise in diabetic retinopathy diagnosis. Understanding these differences provides valuable insights into the real-world applicability of AI screening.

## Results

### Selection and characteristics of eligible studies

**Fig. 1:**
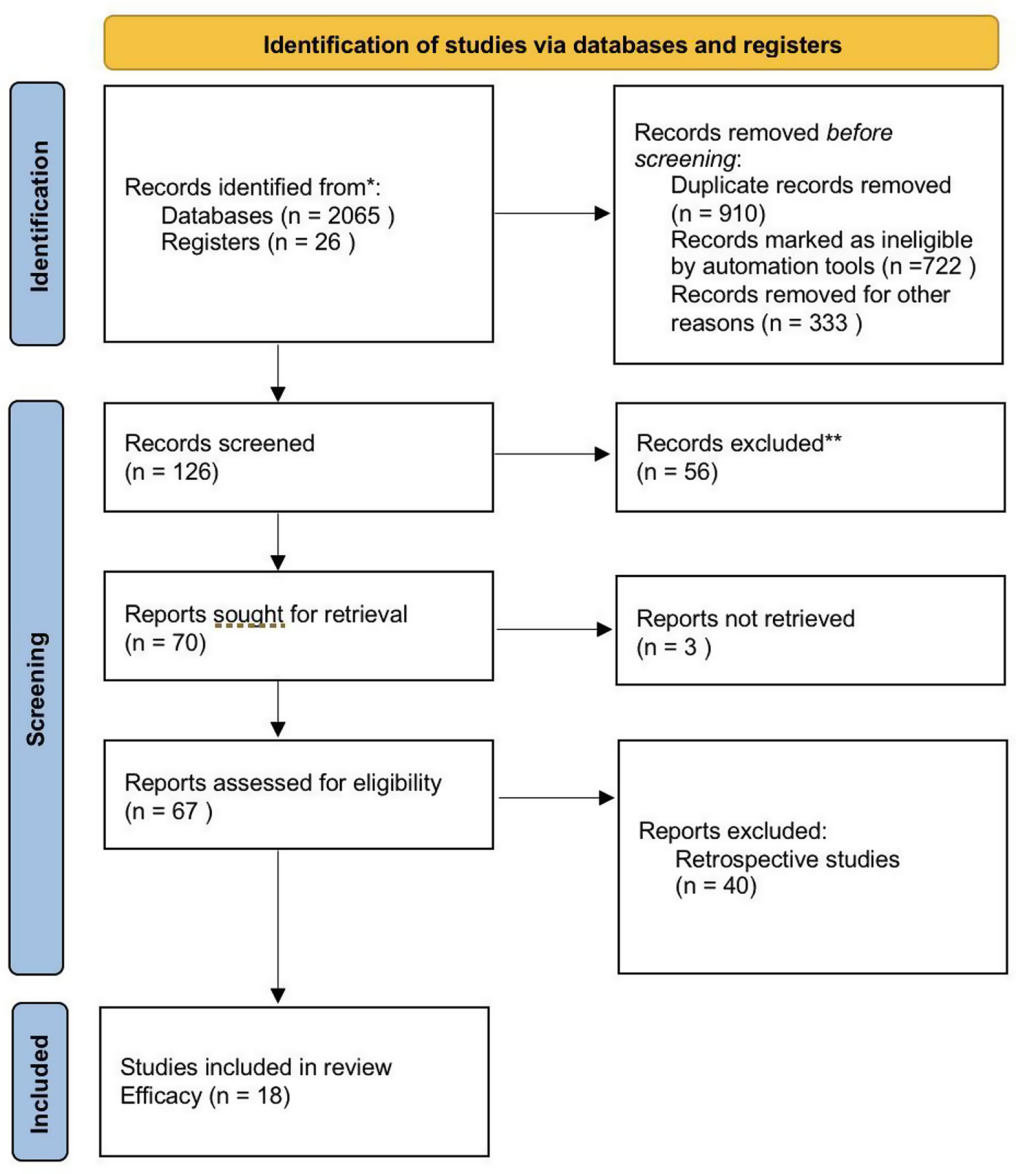
Flow diagram of literature selection

A flowchart of the literature search and study selection process is presented in Fig. 1. The study selection process involved a systematic search of databases and registers, during which a total of 2065 records were identified from databases and 26 from registers. After removing 910 duplicate records, 722 records were marked as ineligible by automation tools, and an additional 333 records were eliminated for various other reasons, resulting in 126 records being screened. Out of these, 56 records were excluded. Subsequently, 67 reports were assessed for eligibility; 40 of these were retrospective studies, leading to 18 studies ultimately included in the review. The eligible studies included in this systematic review exhibited diverse characteristics, contributing to a comprehensive understanding of the research question. A total of 18 studies were reviewed, comprising randomized controlled trials, cross-sectional, and prospective studies. The studies were conducted in various settings, including general population, hospitals and community clinics across multiple countries, such as the United States of America, and China. In addition, two of the studies used OCT for imaging while 16 studies captured the retina only by a fundus image camera. The included studies assessed DR and ME for the screening process and 9 of them only assessed DR. We examined the diagnostic AI utilized by each study for detecting DR and ME, as well as the quality of the images, the geographical areas where the studies were conducted, and the sample sizes involved (Table 1).

**Table 1:** Summary of the data obtained from the included studies

Author	Year	Country	Sample size (patient)	Eye disease	Reference standard (RS)	SN and SP of (RS)	OCT or funds image	AI system	Country of AI system	SN and SP of AI
Lim JI et al. [6]	2022	US	521	DR and ME	General ophthalmologists and retina specialists	Retina specialists: SN 59.5%, SP 98.9% General ophthalmologists: SN 20.6%, SP 99.8%	fundus image	EyeArt	US	SN: 96.4%, SP: 88.4%
Zhang Wf et al. [7]	2022	China	630	DR and ME	Ophthalmologists	N/A	Funds image	EyeWisdom V1	China	SN: 98.23% SP: 74.45
Liu R et al. [8]	2022	China	600	DR and ME	Retina specialists	N/A	OCT and fundus image	Faster R-CNN	China	?
Li N et al. [9]	2021	China	1147	DR	Retina specialist	N/A	Funds image	VoxelCloud	China	SN:85.1% SP:95.6%
Dow ER et al. [10]	2023	US	1222	DR	Retina specialists	SN: 69.5% SP: 96.9%	Funds image	IDx-DR	Us	SN: 95.5% SP: 60.3%
Vebraak Fd et al. [11]	2019	?	1425	DR	Retina specialists	N/A	Funds image	IDx-DR-EU-2.1	The Netherlands	SN: 79.4% SP: 93.8%
Wintergerst Mwm et al. [12]	2021	Germany	75	DR and ME	Trained graders and ophthalmologist experts	SN: 67% SP:74	Funds image	EyeArt version 2.1.	US	SN: 100% SP: 98
Abràmoff Md et al. [13]	2018	US	819	DR and ME	Trained grader	N/A	OCT and fundus	IDx-D	US	SN: 87.2% SP: 90.7
Quellec [14]	2019	France	164,660 (procedures)	DR	Certified ophthalmologists	N/A	Funds image	OphthAI	France	SN:99% SP:90.2
Wang Y et al. [15]	2021	China	4900	DR	Trained graders and licensed and senior ophthalmologists	N/A	Funds image	DLA based on inceotion -V3 convolutional neural networks	China	SN: 97% and SP: 87.9%
Baget-Bernaldiz M et al. [16]	2021	Spain	7164	DR	Retina specialists	N/A	Funds image	DLA built on convolutional neural networks.	Spain	SN: 97.92% SP:99.91
Nunez do Rio JM et al. [17]	2022	India	11,199	DR	Trained optometrists and ophthalmologists	N/A	Funds image	Zeiss VISUHEALTH-AI DR	Singapore	SN: 72.08% SP: 85.65
Gulshan V et al. [18]	2019	India	3049	DR and ME	Trained graders and retinal specialist	Retinal Specialist (Aravind): SN: 89.8% Sp: 83.5% Retinal Specialist (Sankara): SN: 73.4% SP: 98.7% Trained Grader (Aravind): SN: 75.7% Sp: 94.2% Trained Grader (Sankara): SN: 84.2% Sp: 98.6	Funds image	Automated DR grading system based on deep learning algorithms	USA	?
Rogers T.W et al. [19]	2020	Mexico	5752	DR	Board od experts	N/A	Funds image	Pegasus, Visulytix Ltd., U	UK	SN:81.6% SP:81.7
Scheetz J et al. [20]	2021	China	203	DR	NHS-certified retinal grader	N/A	Funds image	?	Australia	SN:96.9% SP:87.7
Zhang Y et al. [21]	2020	China	4726	DR and ME	Ophthalmologis	N/A	Funds image	VoxelCloud	China	SN: 83.3%. SP: 92.5
Wongchaisuwat et al. [22]	2021	Thailand	Nidek: 3515 Eidon:2663	DR and ME	Retinal experts	N/A	Funds image by Nidek and Eidon cameras	DL based on convolutional neural networks	Thailand	SN of AI: Nidek: 93% Eidon: 88% SP of AI: Nidek: 91% Eidon: 85
Ming s et al. [23]	2021	China	193	DR	Retinal experts	N/A	Funds image	China	Eye wisdom	SN: 83.3% SP: 97.9%

### Quality assessment

**Fig. 2:**
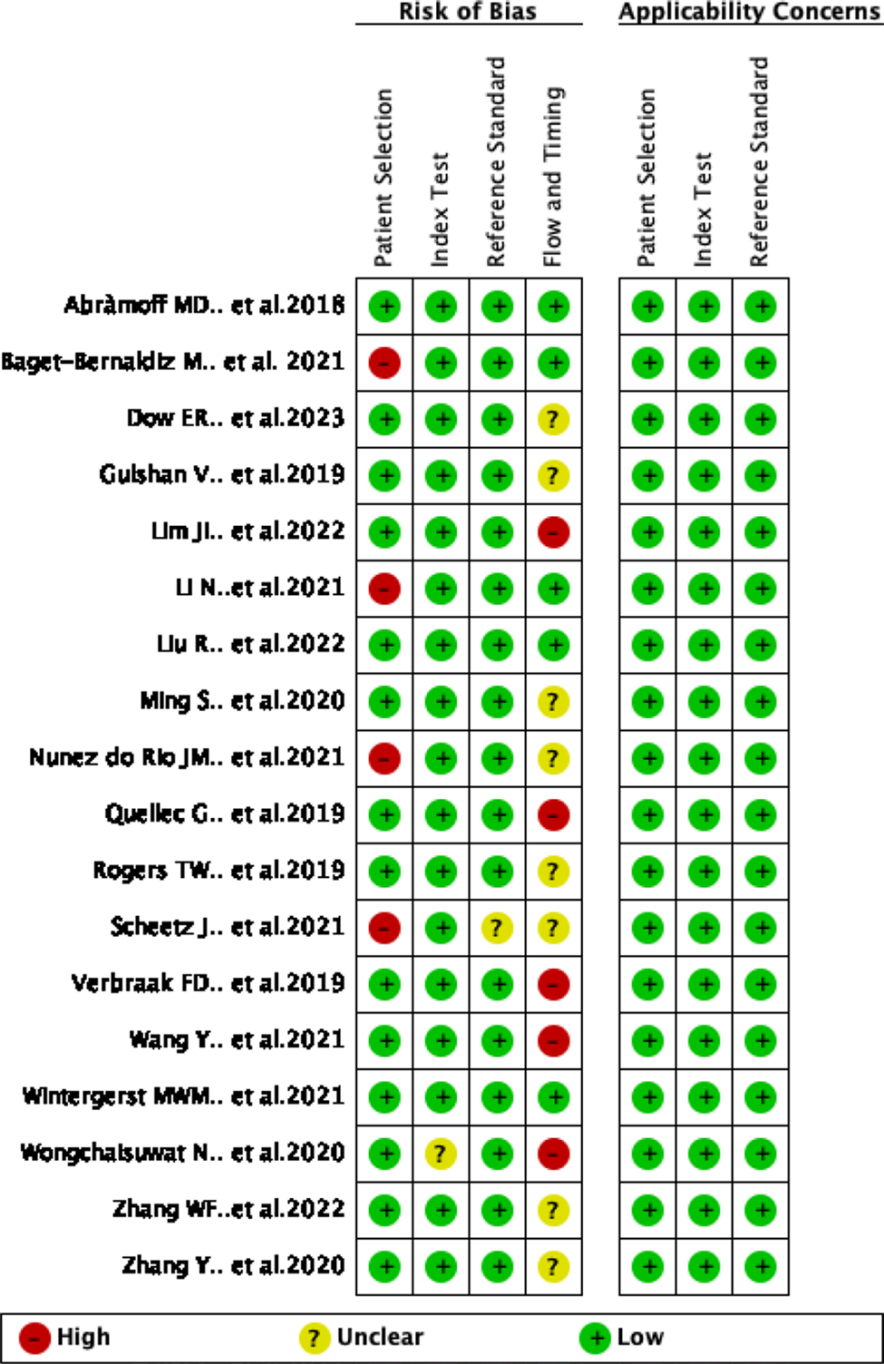
QUADAS-2 risk of bias and applicability concerns summary plot

**Fig. 3:**
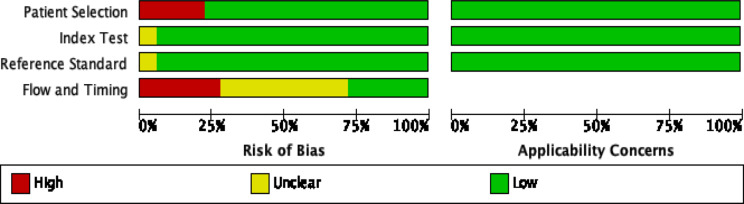
QUADAS-2 risk of bias and applicability concerns bar plot

Figures 2 and 3 show the summary chart and bar chart for quality assessment of the included studies. Out of the studies included, 4 studies, Baget-Bernaldz et al.,2021; LI et al., 2021; Nunz do rio et al., 2021; and scheetz et al., 2021 had high risk of bias for patient selection. Out of the 18 studies, Wonngchaisuwat et al., 2020 had unclear risk of bias for the index testing. 1 study, scheetz et al., 2021 reported unclear information for the establishment and blinding of reference grading. 13 out of the 18 studies performed poorly in the time and flow evaluation. All studies showed low concerns for applicability of patient selection, index testing, and reference grading.

### Threshold analysis and heterogeneity test

**Fig. 4:**
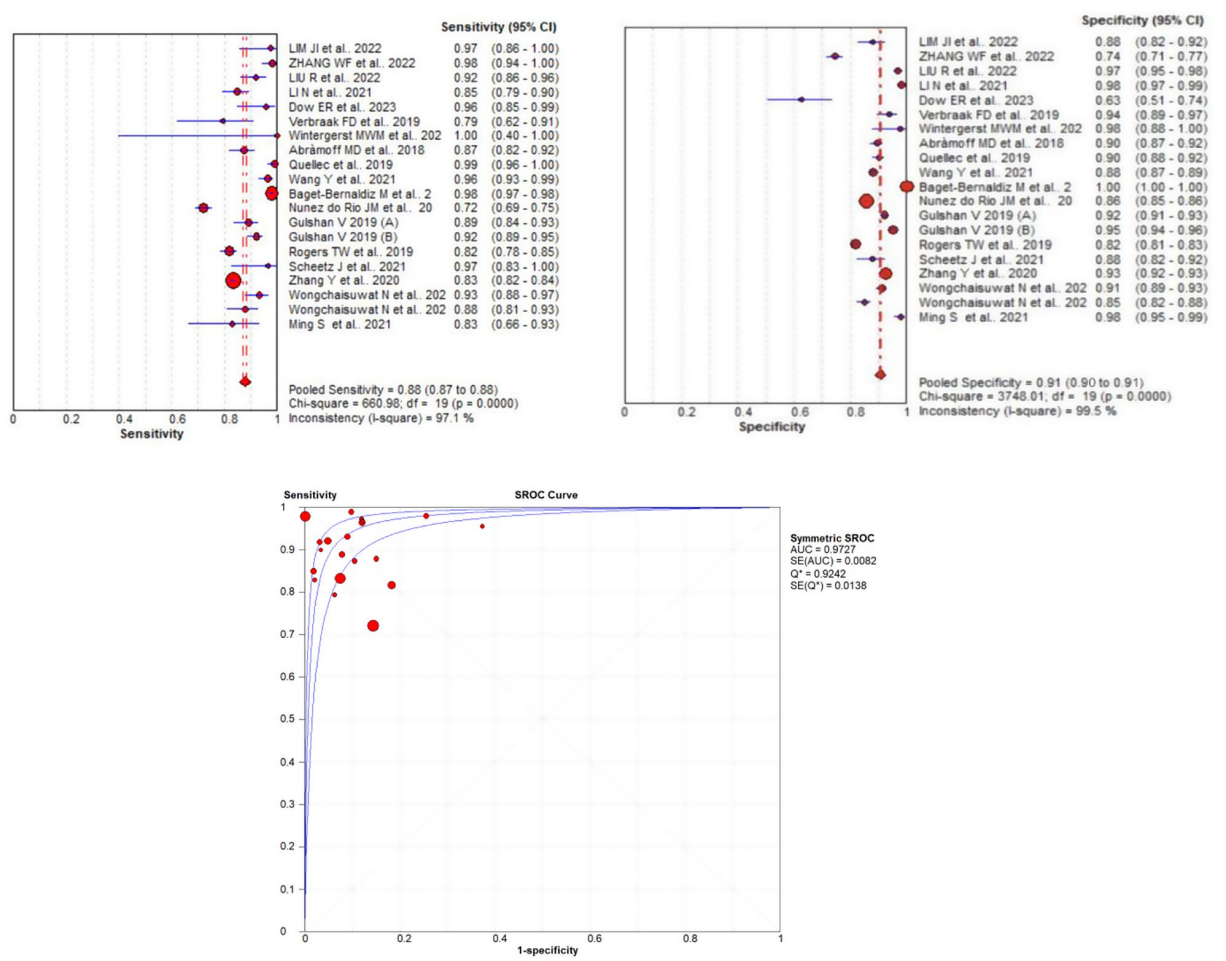
Results of meta-analysis and forest plots of Artificial Intelligence devices. (**A**) Forest plot of pooled Se. (**B**) Forest plot of pooled Sp. (**C**) Summary reciever operating characteristics (SROC) plot

To investigate the presence of a threshold effect, we calculated Spearman’s correlation coefficient between sensitivity and specificity. The correlation coefficient was − 0.45, suggesting a moderate threshold effect across the studies included. An asymmetry was also observed in the summary ROC curve, which indicates that diagnostic performance may be influenced by the threshold levels used in different studies. This suggests that different studies may have applied varying diagnostic thresholds, potentially affecting the balance between sensitivity and specificity. We assessed heterogeneity across the included studies using the I² statistic and Cochran’s Q test. Cochran’s Q test was significant for both sensitivity and specificity (*p* < 0.05), the SE, SP, LR+, LR-, and DOR are all shown in Table 2. The differences in study design, patient populations, and diagnostic thresholds likely contribute to the heterogeneity observed in sensitivity and specificity estimates (see Fig. 4).

**Table 2:** The combined predictive value of all included studies

Coefficient	Merge Value	95% CI	I²
**For AI:**			
Sensitivity	0.877	0.870–0.884	97%
Specificity	0.906	0.904–0.908	99%
DOR	153.79	79.622–297.05	98%
LR+	12.561	8.601–18.343	99%
LR-	0.094	0.065–0.137	96%
**For Doctors**:			
Sensitivity	0.751	0.736–0.766	97%
Specificity	0.941	0.936–0.946	99%
DOR	82.250	42.090–160.73	92%
LR+	20.971	8.514–51.652	98%
LR-	0.310	0.165–0.582	99%

### Synthesis of results

The included data on AI systems and doctors in screening diabetic retinopathy were analyzed using MetaDiSc software (version 1.4). The AI-based screening systems demonstrated high diagnostic accuracy, with pooled sensitivity and specificity of 0.877 (95% CI: 0.870–0.884) and 0.906 (95% CI: 0.904–0.908), respectively. Additional diagnostic performance metrics, including the diagnostic odds ratio (DOR), and likelihood ratios (LR + and LR-) are summarized in Table 2.

For doctors, the pooled sensitivity and specificity were 0.751 (95% CI: 0.736–0.766) and 0.941 (95% CI: 0.936–0.946), respectively. Additional performance metrics, including the diagnostic odds ratio (DOR), and likelihood ratios (LR + and LR-) are summarized in Table 2.

**Fig. 5:**
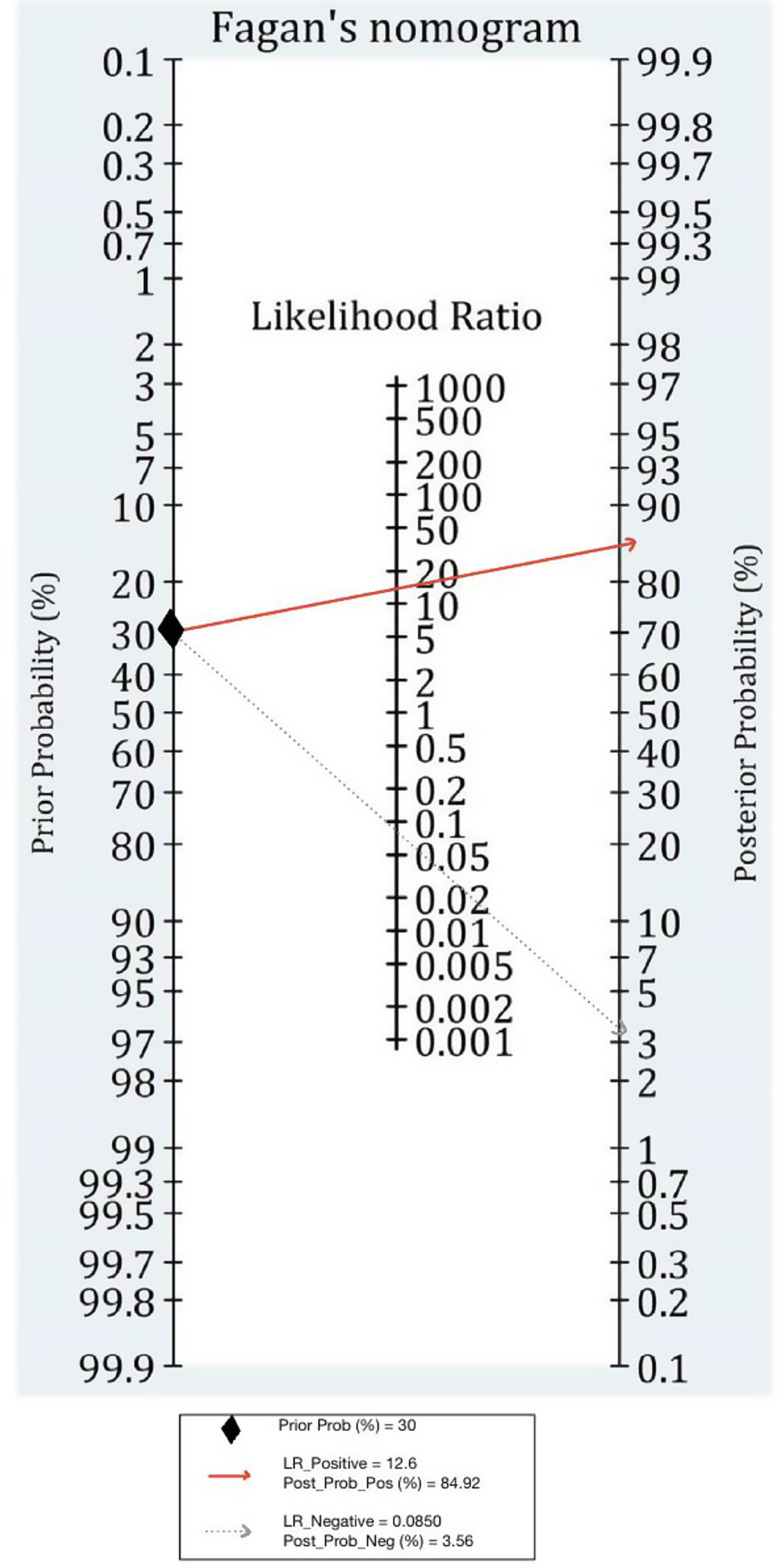
Fagan nomogram of artificial intelligence (AI) for the diagnosis of diabetic retinopathy (DRP)

The Fagan nomogram analysis demonstrated the clinical utility of AI-based DR screening. If a patient tests positive for DR using AI, the post-test probability of truly having the disease increases to 84.92%, indicating that AI effectively enhances disease detection. Conversely, a negative AI result reduces the probability of disease presence to just 3.56%, highlighting AI’s potential to rule out DR with confidence. These findings reinforce the role of AI in triaging patients, allowing ophthalmologists to focus on high-risk cases requiring further evaluation (Fig. 5). Subgroup analyses based on factors such as imaging modality, and doctor expertise revealed further insights into diagnostic performance. These results are shown in Table 3.

**Table 3:** Results of subgroup analysis

Subgroup	Pooled Sensitivity (95% CI)	Pooled Specificity (95% CI)	AUC	DOR (95% CI)	Adjusted DOR (ADOR)/*p* value
Fundus Images	0.93 [0.87, 0.95]	0.92 [0.86, 0.96]	0.92	135.02 [82.72,220.39]	-
OCT + Fundus images	0.93 [0.84, 0.93]	0.94 [0.82, 0.98]	0.92	135.17 [87.82,208.04]	-
Adjusted DOR (ADOR)	-	-	-	-	1.00 [0.52, 1.92] / 0.997
Retina Specialist	0.75 [0.64, 0.83]	0.95 [0.82, 0.99]	0.85	56.68 [38.52, 84.36]	-
Ophthalmologist	0.62 [0.38, 0.81]	0.99 [0.96, 1.00]	0.80	119.76 [89.72,290.81]	-
Adjusted DOR (ADOR)	-	-	-	-	0.47 [0.17, 0.72] / 0.0039

### Meta regression and sensitivity analysis

**Fig. 6:**
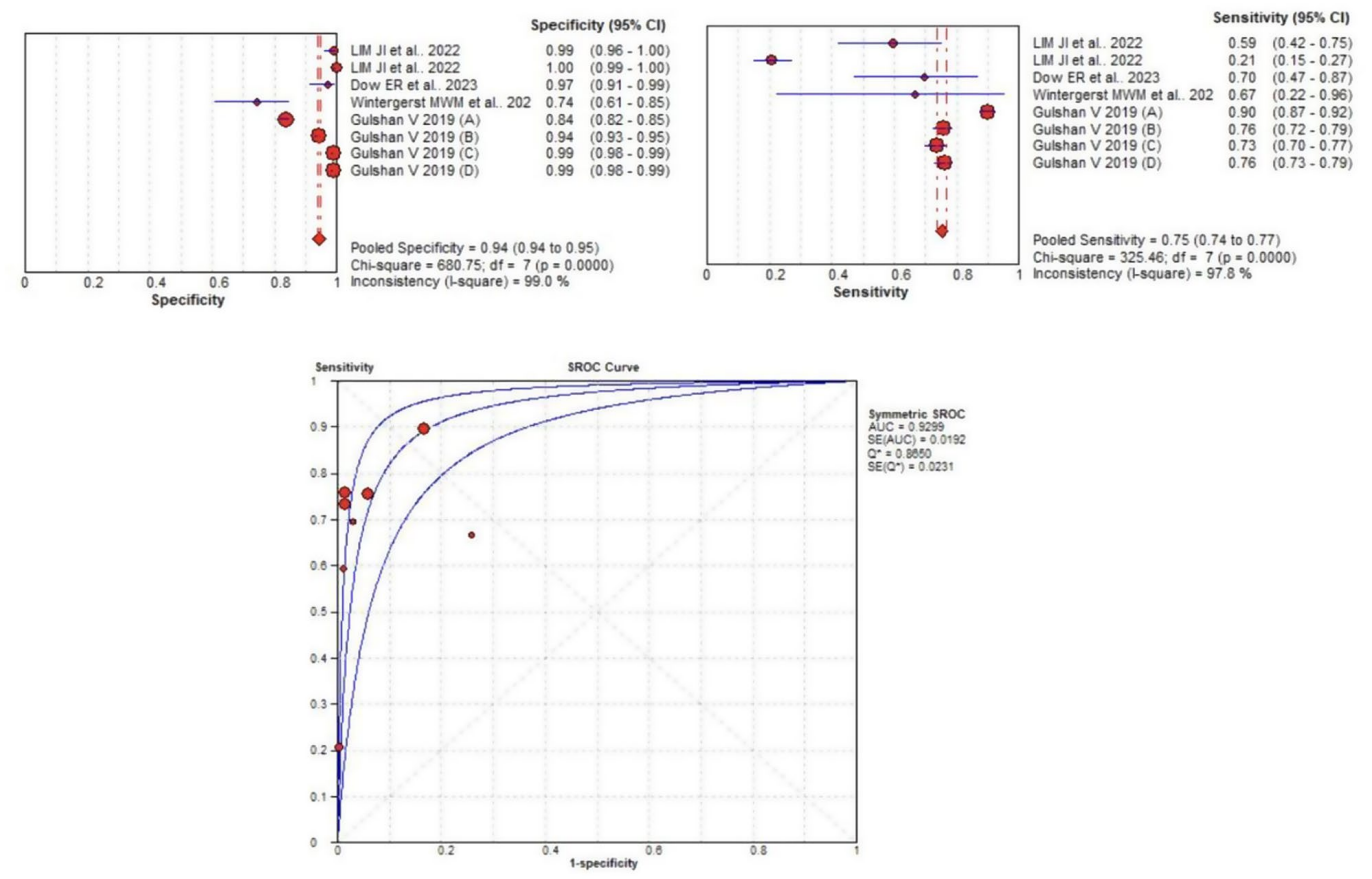
Results of meta-analysis and forest plots of doctors. (**A**) Forest plot of pooled Se. (**B**) Forest plot of pooled Sp. (**C**) Summary receiver operating characteristics (SROC) plot

To explore the sources of heterogeneity, we performed a meta-regression analysis using the MetaDiSc software (version 1.4), evaluating the potential influence of covariates such as imaging modality (fundus vs. OCT) and doctor expertise (retina specialist vs. ophthalmologist). The analysis revealed that both the doctor and AI datasets exhibited high levels of heterogeneity, with I² values exceeding 98% for doctors and 99% for AI systems. This heterogeneity suggests substantial variability in study methodologies, including differences in AI model architecture, image quality, patient populations, and diagnostic thresholds used to classify diabetic retinopathy (see Fig. 6).

High heterogeneity affects the interpretation of pooled results by potentially exaggerating or underestimating AI’s diagnostic performance in different settings. One key factor contributing to heterogeneity is the variation in AI training datasets—models trained on diverse populations may generalize better than those trained on homogeneous datasets. Additionally, differences in study inclusion criteria (e.g., DR severity grading) and reference standards for diagnosis could influence pooled estimates.

Despite this heterogeneity, sensitivity analyses confirmed the robustness of the results. The overall pooled estimates remained significant across different subgroup exclusions, indicating that AI consistently demonstrated strong diagnostic performance across multiple study conditions.

### Publication bias

**Fig. 7:**
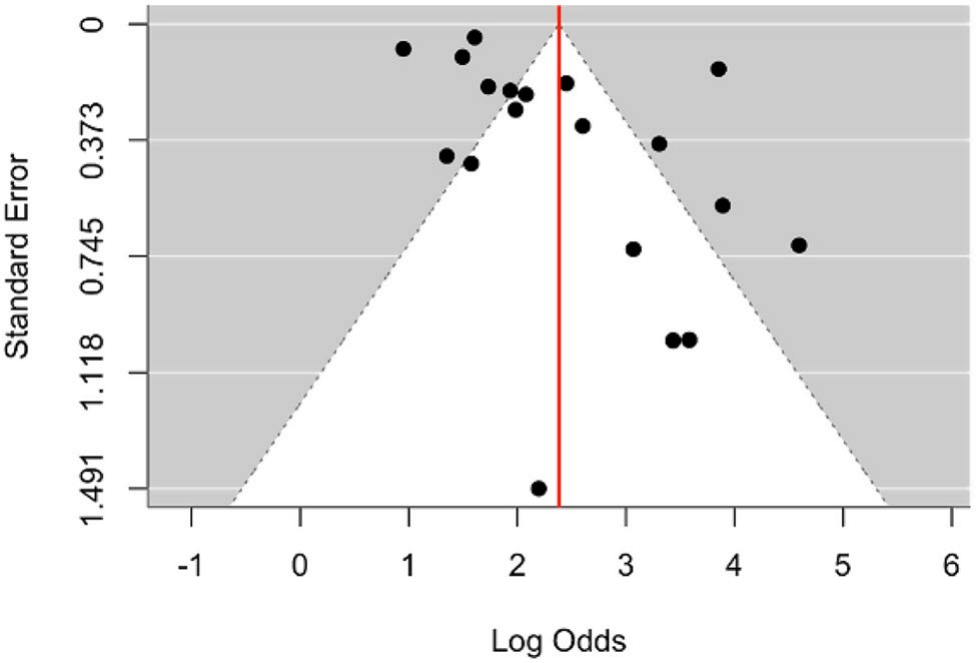
Funnel plot

**Fig. 8:**
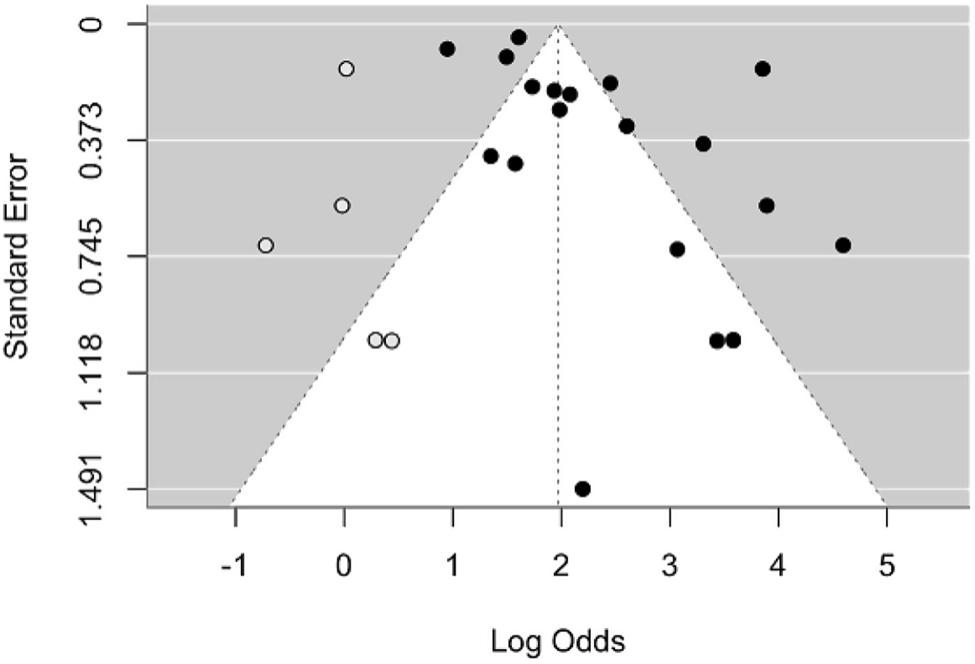
Trim-and-Fill funnel plot

Publication bias was assessed through visual inspection of a funnel plot and statistical tests, including Egger’s test. The funnel plot (Fig. 7) demonstrated asymmetry, suggesting the presence of potential publication bias. Specifically, there was a noticeable lack of studies on the left side of the funnel, indicating that smaller studies with non-significant or smaller effect sizes may be underrepresented in the analysis. This was further supported by Egger’s test, which produced a statistically significant result (t = 2.1400, *p* = 0.0472), confirming asymmetry and the likelihood of publication bias. A trim-and-fill plot (Fig. 8) was then made to adjust for the publication bias.

## Discussion

Over the past years artificial intelligence has revolutionized many fields including healthcare. AI can be utilized as a screening tool aiding in the early detection of many conditions such as diabetic retinopathy [24]. AI models, especially those based on deep learning and machine learning techniques, have demonstrated effectiveness in screening for diabetic retinopathy.

In our systematic review and meta-analysis, we included 18 studies with a total of (214,463) patients. We assessed the effectiveness of AI compared to human graders (ophthalmologists and retina specialists) by analyzing the sensitivity, specificity, and diagnostic odds ratios (DOR). Our results reveal that AI shows strong diagnostic performance. However, there was some notable heterogeneity among the studies and concerns for publication bias.

One of our key findings was the high heterogeneity among the included studies, with I² values frequently surpassing 97% in numerous analyses. This heterogeneity could possibly arise from differences in imaging techniques, AI algorithms, and study demographics [25, 26]. Although we performed a sub-group analysis to discover the source of this considerable variability among the studies, the main source remains unclear. One of the potential sources of this heterogeneity is the variable threshold among the studies. Different studies could be using different cut-points to detect and categorize diabetic retinopathy. As these thresholds fluctuate between the studies, the false positive and false negative results fluctuate as well. Another contributing factor is the diversity in AI training. AI models trained on a diverse dataset will generalize differently compared to other models trained on a more uniform dataset. AI models that are trained on a more uniform dataset, for example, will encounter some challenges in the diverse clinical setting Whereas another AI model trained on a diverse dataset will show a better performance. Notably, a research conducted by Wu et al. indicated that machine learning models trained on a diverse dataset show more efficacy in clinical situations [27]. These differences in AI training create a varying diagnostic effect leading to high heterogeneity levels [28]. Moreover, the use of different imaging techniques such as fundus photography and OCT may have contributed to this significant heterogeneity.

To explore the source of heterogeneity further, we performed subgroup analyses based on clinician (ophthalmologist versus retina specialist) and imaging technique (fundus image only versus fundus image + OCT). The results revealed that AI interestingly outperformed clinicians. AI had a pooled sensitivity of 0.877 and specificity of 0.906 while retina specialists had sensitivity of 0.750 and specificity of 0.949. It is worth noting that general ophthalmologists had lower sensitivity compared to retina specialists which indicates that AI can considerably outperform general ophthalmologists in detecting diabetic retinopathy. This aligns with the findings of previous studies. A study by Gulshan et al. found that in the screening of diabetic retinopathy, AI could outperform general ophthalmologists as AI is consistent in analyzing and identifying minute patterns in large datasets compared to human experts [29].

Regarding imaging techniques, using fundus images and OCT did not show significant difference compared to using fundus images alone. This indicates that AI is useful in the screening of diabetic retinopathy over a wide range of settings, especially in areas with limited resources where OCT may not be readily available. Previous research done by Abramoff et al. and Gulshan et al. show that AI trained on fundus image alone could achieve comparable results to those of clinicians [13, 29]. For instance, Gulshan et al. found that AI trained on fundus images achieved a sensitivity of 90.3% and a specificity of 98.1% in detecting referable diabetic retinopathy without the need for the advanced technology of OCT. This means that AI can recognize important indicators of diabetic retinopathy such as, microaneurysms, hemorrhages, and exudates based solely on fundus image making it highly efficient in broad clinical use [29].

This study addresses critical gaps in the literature. It is the first to include a detailed subgroup analysis based on imaging modality and clinician expertise. The efficacy of AI screening using fundus photography versus OCT has not been discussed before. Moreover, this study uniquely compares AI versus clinicians with different levels of expertise including general ophthalmologist and retina specialist. This approach allows for a deeper understanding of how AI performance varies across different clinical scenarios, and whether more advanced imaging modalities or higher levels of expertise are required for efficient screening. Furthermore, our meta-analysis includes recently published studies, ensuring that the findings reflect the most current data available in the field.

The use of AI in DR screening is promising in terms of efficiency. Yet, the question whether using AI is cost effective or not is an important consideration. There is conflict in the literature, and it might be attributable to some factors such as geography and deployment of the strategy [30]. Artificial intelligence has demonstrated significant cost-effectiveness in screening for diabetic retinopathy. A Markov model analysis conducted in rural China evaluated the economic viability of AI screening compared to no screening and ophthalmologist-led methods. The study revealed that AI screening increased quality-adjusted life years (QALYs) by 0.16 at an incremental cost of $180.19 compared to no screening. In contrast, ophthalmologist-led screening was less effective and more expensive. The incremental cost-effectiveness ratio (ICER) for AI screening was $1,107.63, which was well below the cost-effectiveness threshold of one to three times the per capita GDP, affirming its feasibility in resource-constrained environments [31].

Furthermore, a systematic review of AI-based DR screening systems highlighted their potential to scale effectively, reducing screening costs and improving accessibility, particularly in underserved regions. Notably, AI systems reduce reliance on ophthalmologists for initial DR detection, enabling healthcare systems to reallocate resources to treatment and follow-up care, thereby improving cost-effectiveness across the care pathway [30]. By combining accurate diagnosis with reduced costs, AI-based DR screening offers a scalable solution to alleviate the economic burden of vision loss, making it an invaluable tool in achieving global health equity [30, 31].

Despite the promising results, our study has several limitations that should be acknowledged. First, the high heterogeneity across studies limits the generalizability of the findings. Second, the presence of publication bias, as indicated by the asymmetry in the funnel plot and Egger’s test, suggests that smaller or non-significant studies may be underrepresented in the analysis. Although we applied the trim-and-fill method to adjust for missing studies, publication bias remains a concern that could have influenced the pooled estimates. Third, In the meta regression, we did not further analyze patient information, such as age, sex, and duration of the disease, which may be a source of heterogeneity and need further studying. Finally, only English studies were included, which may cause a bias due to the lack of literature in other languages.

## Conclusion

In conclusion, AI systems have demonstrated strong diagnostic performance in detecting diabetic retinopathy, with sensitivity and specificity comparable to or exceeding traditional clinicians. Their ability to outperform non-specialist clinicians highlights the promise of integrating AI into clinical practice, particularly for large-scale populations. As AI technology continues to improve and becomes more cost-effective, its integration could significantly enhance the early detection and treatment of DR, ultimately reducing the global burden of blindness. Emphasizing the practical application of AI in clinical settings will be vital for realizing its full benefit. Furthermore, future research should aim to standardize AI evaluation metrics and dataset diversity to reduce variability in future meta-analyses.

## Electronic supplementary material

Below is the link to the electronic supplementary material.

Supplementary Material 1

## Data Availability

All data generated or analyzed during this study are included in this published article.
