# Supplementary material for: The efficacy of artificial intelligence in diabetic retinopathy screening: a systematic review and meta-analysis
Source: Int J Retina Vitreous. 2025 Apr 22;11:48. doi: 10.1186/s40942-025-00670-9 (PMC12012971; doi:10.1186/s40942-025-00670-9)
Supplement: Supplementary file 1 — Supplementary Material 1 [file 40942_2025_670_MOESM1_ESM.docx]

| **Database** | **Search String** |
| --- | --- |
| **PubMed Medline** | ("diabetic retinopathy"[MeSH] OR "diabetic eye disease" OR "retinal screening") AND ("artificial intelligence"[MeSH] OR "deep learning" OR "machine learning" OR "neural networks" OR "computer-aided diagnosis") |
| **Cochrane Central** | ("diabetic retinopathy" OR "diabetic eye disease" OR "retinal screening") AND ("artificial intelligence" OR "deep learning" OR "machine learning" OR "neural networks" OR "computer-aided diagnosis") |
| **ScienceDirect** | TITLE-ABS-KEY("diabetic retinopathy" OR "diabetic eye disease" OR "retinal screening") AND TITLE-ABS-KEY("artificial intelligence" OR "deep learning" OR "machine learning" OR "neural networks" OR "computer-aided diagnosis") |
| **Web of Science** | TS=("diabetic retinopathy" OR "diabetic eye disease" OR "retinal screening") AND TS=("artificial intelligence" OR "deep learning" OR "machine learning" OR "neural networks") |
